# Distinct spatial scale sensitivities for early categorization of faces and places: neuromagnetic and behavioral findings

**DOI:** 10.3389/fnhum.2013.00091

**Published:** 2013-03-21

**Authors:** Bhuvanesh Awasthi, Paul F. Sowman, Jason Friedman, Mark A. Williams

**Affiliations:** Waisman Center, University of Wisconsin-MadisonWI, USA; Department of Cognitive Science, ARC Centre of Excellence in Cognition and its Disorders, Macquarie UniversitySydney, NSW, Australia; Department of Physical Therapy, Tel Aviv UniversityTel Aviv, Israel

**Keywords:** spatial frequency, face perception, place perception, M170

## Abstract

Research exploring the role of spatial frequencies in rapid stimulus detection and categorization report flexible reliance on specific spatial frequency (SF) bands. Here, through a set of behavioral and magnetoencephalography (MEG) experiments, we investigated the role of low spatial frequency (LSF) (<8 cycles/face) and high spatial frequency (HSF) (>25 cycles/face) information during the categorization of faces and places. Reaction time measures revealed significantly faster categorization of faces driven by LSF information, while rapid categorization of places was facilitated by HSF information. The MEG study showed significantly earlier latency of the M170 component for LSF faces compared to HSF faces. Moreover, the M170 amplitude was larger for LSF faces than for LSF places, whereas the reverse pattern was evident for HSF faces and places. These results suggest that SF modulates the processing of category specific information for faces and places.

## Introduction

Perception of visual objects in our environment is carried out in several steps, in a hierarchical manner. Amongst the wide variety of stimuli, faces are a special class of biological stimuli that are recognized rapidly owing to their obvious survival value (Thorpe et al., 1996; Honey et al., 2008; Crouzet et al., 2010). Perception of faces is carried out in a more configural or holistic manner than other categories, such as places (Young et al., 1987; Maurer et al., 2002). The specific mechanisms supporting configural and part-based information are investigated using behavioral and neuroimaging methods. In particular, it remains to be determined how stimuli such as faces, places and objects are coded as distinct categories in the visual hierarchy for higher-level abstract processing.

Early work suggests that perceptual processing occurs initially at the most global level (Navon, 1977). According to Hughes et al. (1990), this global dominance effect may stem from low spatial frequency (LSF) information. In accordance with the coarse-to-fine approach, the perceptual system prioritizes processing of coarse information over fine-grained information in visual stimuli for efficient detection, categorization and identification (Marr, 1982; Ginsburg, 1986; Parker and Costen, 1999). Spatial filtering is known to occur early on (Wilson and Bergen, 1979; Ginsburg, 1986) and a variety of tasks are reported to be affected by this filtering, such as edge detection (Marr and Hildreth, 1980; Watt and Morgan, 1985) and motion perception (Morgan, 1992). As an alternate to the fixed coarse-to-fine approach, a diagnostic approach has been proposed that argues against the uni-directional inputs for categorization processes (Schyns and Oliva, 1994, 1999; Schyns, 1998; Morrison and Schyns, 2001). In this context, the flexible usage approach accords that different spatial scales should facilitate categorization of different visual stimuli, such as faces, places and objects in a differential manner.

Several behavioral (Goffaux et al., 2005; Goffaux and Rossion, 2006) and neuroimaging (Vuilleumier et al., 2003; Pourtois et al., 2005; Rotshtein et al., 2007) studies support the differential role of spatial frequency (SF) in various aspects of face processing. Configural properties of stimuli tend to be better represented by coarser scales and a bias toward LSF (as opposed to high spatial frequency (HSF)) might support faster category-level judgement for faces. Examining the role of low and high SF in configural and featural processing of faces, Goffaux et al. (2005) reported a strong performance advantage using LSF information (<8 cycles/face, cpf) for configural processing and HSF (>32 cpf) support for featural processing. LSF information is reported to be sufficient for familiarity judgement and famous faces can easily be recognized using coarse-scale blurred information (Sinha, 2002; Sinha et al., 2006). In contrast, Halit et al. (2006) demonstrated that faces containing both HSF and LSF information are detected faster and more accurately than LSF faces and argued for the importance of HSF information in the early stages of face perception.

Humans are also reportedly quick at detecting briefly viewed natural scenes and other -face stimuli (VanRullen and Thorpe, 2001). Rapid detection of scenes involves an interaction of bottom-up and top-down processes and the efficient categorization is attributed to quick processing via magnocellular pathways (Delorme et al., 1999, 2000). Work by Oliva and Schyns (1997) discussed the influence of LSF and HSF in the categorization of scenes and showed that different spatial scales are used depending on the task. Further, Oliva and Torralba (2006) argued that “scene gist” proceeds in a global manner, but does not necessarily rely solely on LSF and involves several bands of spatial frequencies. In contrast, scene categorization was shown to rely on HSF information that aids navigation and identification (Rajimehr et al., 2011).

Using magnetoencephalography (MEG), several studies have examined the M170—a neuromagnetic response that peaks at 130–200 ms after stimulus onset and shows a larger response to faces than to other stimuli (Liu et al., 2000, 2002; Xu et al., 2005; Harris and Nakayama, 2007). The M/N170 (the EEG analogue of M170) has been thought to index configural processing (Bentin et al., 1996; Rossion et al., 2000; Itier et al., 2006; Harris and Nakayama, 2007), while others have argued that M/N170 responds to specific face parts such as the eye region (Schyns et al., 2003; Smith et al., 2007). In an MEG study using spatially filtered faces, Hsiao et al. (2005) reported lesser M170 activation to LSF faces as compared to HSF faces and argued for the importance of feature-based face processing. Similarly, in another MEG study, Harris and Nakayama (2008) reported rapid adaptation of the M170 response to face parts but not to face configuration. Thus, the exact role of M170 in configural vs. featural processing of faces remains controversial and calls for further investigation.

Different frequency cut-offs have been reported to be important for a variety of tasks. Several studies have reported the preferential role of the lowest band of frequencies (2–8 cpf) to be more important in the representation of a global percept of a face (Collishaw and Hole, 2000; Goffaux et al., 2003, 2005; Goffaux, 2009; de Heering et al., 2008). It has also been reported that human observers are not able to utilize information in all the SF bands with equal efficiency and rely more on mid-band, rather than LSF or HSF (Gold et al., 1999; Kornowski and Petersik, 2003). The middle band of frequencies situated around 8–16 cpf, is reported to be important in identity recognition (e.g., Gold et al., 1999; Näsänen, 1999; Tanskanen et al., 2005), while the fine-tuned analysis of local details is based on higher ranges of SF (above 32 cpf; Goffaux and Rossion, 2006). In our study here, rather than an identification task or expression task, we examined category-level judgement using face and building images. Accordingly, we chose to contrast our conditions maximally and selected to use images below 8 cpf as LSF and images above 25 cpf as HSF [adapted from early work by Schyns and Oliva (1994, 1999)].

Recently, using visually guided reaching as a continuous behavioral measure, we explored the relative role of LSF and HSF in hybrid faces and demonstrated interference by LSF information at the periphery (Awasthi et al., 2011a,b). While reaching trajectories provide crucial information about the evolution of the decision-making process, reaction times could not be explored as participants were compelled to initiate movements quickly. Examining cortical responses will provide neural evidence of the role of SF information in face and place categorization. We therefore designed a behavioral reaction time experiment, as well as an MEG experiment to examine LSF and HSF processing of both face and place images, presented briefly at the fovea and at left and right periphery. As configural properties tend to be better represented at coarser scales, LSF information is likely to facilitate rapid categorization of faces. In contrast, part-based detection of sharp edges, required for places, is likely to be facilitated by HSF information.

Face processing is reported to be more efficient at the fovea compared to periphery and has a central field bias (Kanwisher, 2001; Levy et al., 2001), while places are processed relatively more peripherally. However, it has also been shown that HSF channels dominate central vision (De Valois and De Valois, 1988), and LSF channels support peripheral processing. Also, Rousselet et al. (2005) demonstrated that foveal bias for face processing could be eliminated by scaling the stimulus size in accordance with the cortical magnification factor for the primary visual cortex. In the experiments reported here, in addition to the spatial scale contribution to categorization, we also explore this fovea-periphery distinction for faces and places.

## Methods

The Macquarie University Human Research Ethics Committee (HREC) approved the ethical aspects and experimental protocol of this study and subjects gave written, informed consent before participation.

### Stimuli, design, and procedure

**Figure 1:**
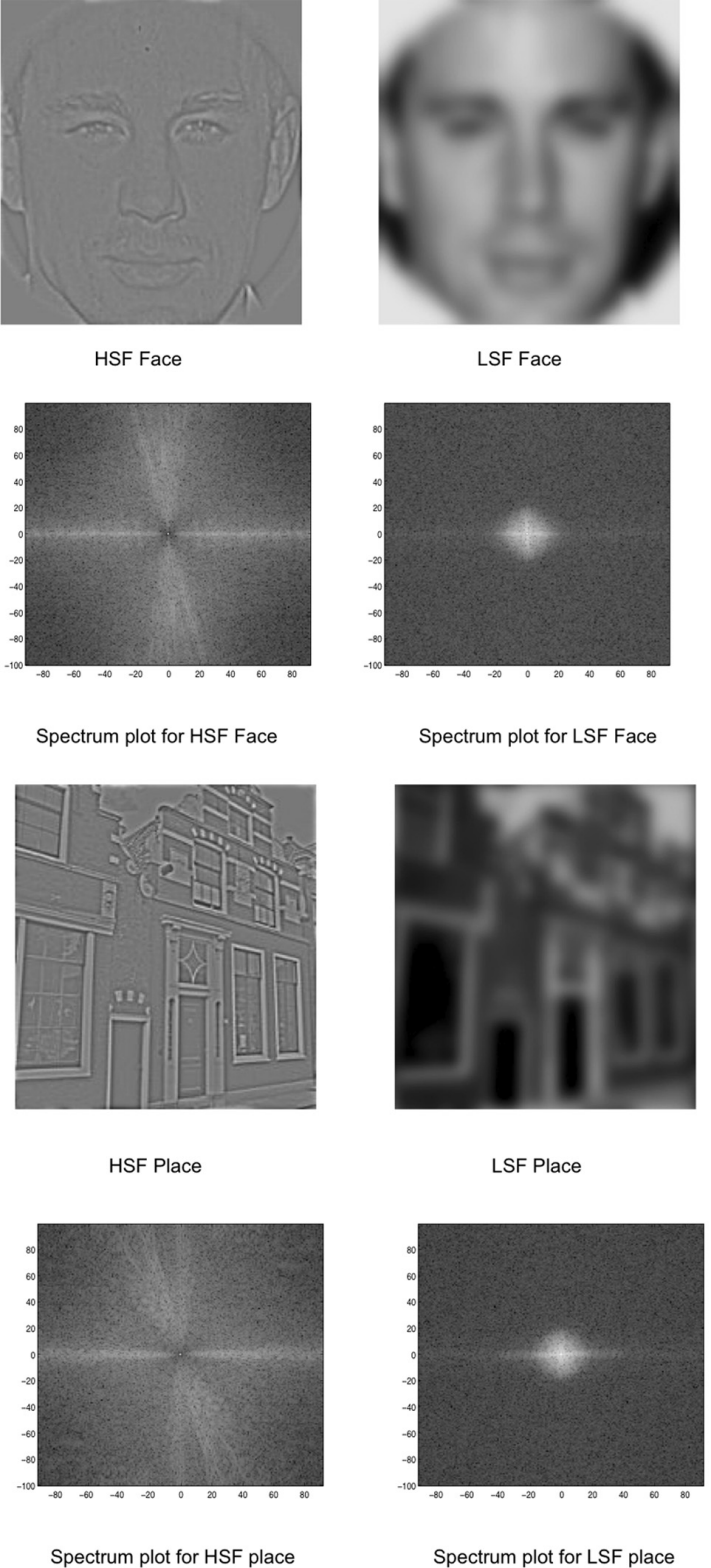
**Stimuli used in the experiment: HSF and LSF versions of faces and places.** The spectrum is displayed as a polar plot, where contrast energy (log) is plotted as a function of spatial frequency (distance from the origin; low-to-high) and orientation (angle).

Images of unfamiliar faces and houses/buildings were collected from the internet and converted to a 256 gray-level scale. The face images had neutral expressions with an equal number of both male and female, young, Caucasian faces in frontal views and direct gaze. Faces were approximately the same width in visual angle and did not vary in size in relation to the image. Both faces and buildings filled the entire image (see Figure 1). There were 80 face and 80 building images.

Using the Matlab-based SHINE (spectrum, histogram, and intensity normalization and equalization) toolbox (Willenbockel et al., 2010), the images were equated for contrast, mean luminance, and exact histogram specifications. After this matching, the images were then filtered for LSF and HSF versions. Using a customized code adapted from Schyns and Oliva (1999), the images were Fourier transformed and low-pass and high-pass Gaussian filters were applied to preserve LSF (below 8 cycles/image) and HSF (above 25 cycles/image) information in each image. The Fourier amplitude spectrum (displayed as a polar plot, showing contrast energy) of the corresponding images is also shown in Figure 1.

### Behavioral experiment

#### Subjects

Fifteen right-handed subjects (8 females; minimum age: 18 years, median age: 24 years, maximum age: 44 years, mean age: 26.6 years, *SD* = 7.1) were recruited from the Macquarie University community. They were paid $15/h for participation which is the standard participant payment rate approved by the HREC. Participants' vision status (normal or corrected-to-normal) was self-reported. They were asked to wear spectacles/contact-lenses if they used them on a regular basis. Subjects were required to categorize the target as either face or place by pressing appropriate buttons with their fingers. We used a custom-built button box that was connected to a measurement computing data acquisition (DAQ) card (PCI-DIO-24). The order of buttons was interchanged in a counterbalanced fashion for all subjects.

Presentation software (Neurobehavioral Systems) was used to present the stimuli. The stimuli had a mean width of 2.7° visual angle and were presented 21.7° from fixation for peripheral conditions. Subjects sat on a -moving, -swiveling chair (at a fixed distance from the screen) in a quiet, dark room at a table with a LCD screen (Philips LCD BDL3221V model) (70 × 39 cm, 1360 × 768 pixels, 60 Hz) positioned approximately 70 cm in front of them. In each trial, subjects were presented with a central fixation cross, followed by one image for 33 ms, at either the fovea or left periphery or right periphery. The stimuli were presented in a pseudo-randomized order without repetition and stimuli type were counterbalanced across runs. Each block consisted of 56 trials. After two blocks of training, twenty experimental blocks were run with adequate breaks in between. Feedback was provided onscreen only during training blocks.

#### Statistics and analysis

As the accuracy rate was remarkably high across all experimental conditions (mean: 93.27%; min accuracy: 90.11%; max accuracy: 96.6%, median: 93.59%), only the correct response trials were used for further analysis. Trials with errors (false hits) and delayed responses (three seconds as response cut-off) were excluded and not analyzed. A multivariate analysis of variance (MANOVA) with target type (face, place), SF (LSF, HSF) and location (fovea, periphery), across three quartile (25, 50 or 75%) was carried out followed by (Tukey's HSD) *post-hoc* comparisons.

### Behavioral results

**Figure 2:**
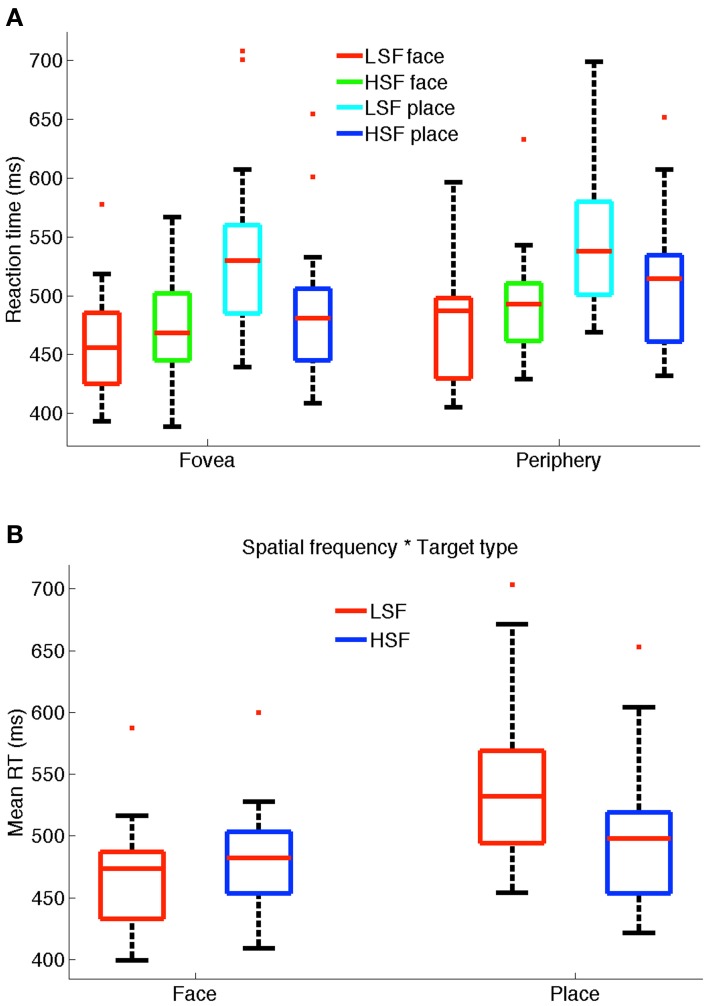
**(A)** Reaction time distribution for faces and places in LSF and HSF conditions for fovea and periphery. The bottom and top of the box show the 25th and 75th percentiles respectively. The whiskers extend to the most extreme value that is less than or equal to 1.5 times the box height. Outliers (values outside the whiskers) are shown by red plus signs. **(B)** Box-plot shows significant two-way interaction between spatial frequency and target type.

Subjects were required to categorize stimuli as face or place via a button press response. We performed a MANOVA on the 25, 50 (median), and 75% RT quartiles. Figure 2A shows the boxplot distribution of reaction times for face and place targets in foveal and peripheral presentations in LSF and HSF conditions for all subjects. The bottom and top of the box show the 25th and 75th percentiles respectively (see figure legend for more details). We report the mean RTs to indicate the magnitude of the differences. Results of the multivariate analysis showed that subjects were significantly faster at the fovea (mean 490 ms) compared to the periphery (mean 507 ms), as shown by a main effect of location [*F*_(3, 12)_ = 22.27, *p* < 0.001]. HSF was significantly faster (mean = 490 ms) than LSF (mean = 506 ms) as shown by a main effect of SF [*F*_(3, 12)_ = 10.81, *p* = 0.001], and faces were categorized significantly faster (mean = 474 ms) than places (mean = 522 ms) as shown by a main effect of target type [*F*_(3, 12)_ = 4.79, *p* = 0.02].

To determine whether these effects were specific to one of the quartiles, we ran univariate ANOVAs on the three quartiles (using a Bonferroni correction). We found the same significant effects for all three quartiles for fovea vs. periphery [25%: *F*_(1, 14)_ = 7.74, *p* = 0.015; 50%: *F*_(1, 14)_ = 39.48, *p* < 0.001; 75%: *F*_(1, 14)_ = 57.67, *p* < 0.001], for LSF vs. HSF [25%: *F*_(1, 14)_ = 15.63, *p* = 0.001; 50%: *F*_(1, 14)_ = 20.73, *p* < 0.001; 75%: *F*_(1, 14)_ = 36.04, *p* < 0.001] and for faces vs. places [25%: *F*_(1, 14)_ = 13.24, *p* = 0.003; 50%: *F*_(1, 14)_ = 14.68, *p* = 0.002; 75%: *F*_(1, 14)_ = 16.33, *p* = 0.001].

Interestingly, LSF facilitated significantly faster categorization of faces compared to places as shown by a significant interaction between SF and target type *F*_(3, 12)_ = 124.04, *p* < 0.001 (see Figure 2B). This was also found using univariate tests of the three quartiles [25%: *F*_(1, 14)_ = 80.12, *p* < 0.001; 50%: *F*_(1, 14)_ = 271.09, *p* < 0.001; 75%: *F*_(1, 14)_ = 175.74, *p* < 0.001]. *Post-hoc* (Tukey's HSD) analysis confirmed faster categorization of LSF faces (mean = 467 ms) than HSF faces (mean = 481 ms) [mean difference = −13.96 (−20.53, −7.39)], whereas HSF places were significantly faster (mean = 499 ms) than LSF places (mean = 545 ms) [mean difference = 45.46 (34.12, 56.80)] at significance level, *p* < 0.001.

No significant interactions were observed for any other combinations, for either the multivariate or univariate tests. We note that the same significant differences were found as a result of the independent variables at the three quartiles. This suggests that the differences in stimuli affected the entire RT distribution and not only, for example, slow or fast responses.

### MEG experiment

#### Subjects and preparation

Fifteen right-handed subjects (9 females; mean age: 26.4 years, *SD* = 5.8) were recruited from the Macquarie University community. They were paid in accordance with the standard participant payment rate ($20/h for MEG experiments) approved by the Human Research Ethics Committee of the University. Participants' vision status (normal or corrected-to-normal) was self-reported. They were asked to wear spectacles/contact-lenses if they used them on a regular basis. Subjects self-reported that they did not have a history of any neurological or psychiatric condition. Subjects for the MEG experiment were different from those for the behavioral experiment.

Before subjects entered the magnetically shielded room for MEG DAQ, their head shape was recorded using a digitizing pen (Polhemus Fastrack, Colchester, VT); approximately 600 randomly selected points were recorded for each subject's head surface. The 3D locations of the five head position indicator (HPI) coils attached to a tightly fitting elastic cap, and the locations of three cardinal landmarks (the nasion and bilateral preauricular points) were also digitized. Each subject's head position in the MEG dewar was measured at the start of each recording block from the five HPI coils. A maximum threshold of 5 mm for any individual coil was set as movement tolerance.

Subjects lay comfortably in the scanner. A back projection system (using an InFocus IN5108 projector) was used to present them with a fixation cross on a screen, followed by the stimulus image (either LSF or HSF face or place) at a foveal or peripheral location for 500 ms with an inter-trial interval of 1500 ms. Subjects were required to press a button when an identical image was repeated twice in a row (one-back task). There were 80 exemplars of each image type. Each block had 48 + 5 repeated (10% one back instances) trials. 20 blocks of trials were presented in one scanning session of about an hour. As a crucial part of task instructions, subjects were required to maintain fixation at the cross throughout the experimental block. All the conditions were randomly interleaved and counterbalanced across runs. A fixation cross was presented constantly at the center to assist fixation throughout the duration of each block. Foveal images were presented at the visual angle of 2.54° while peripheral images were presented 9.87° from fixation for peripheral conditions. The peripheral images were enlarged in accordance with the cortical magnification factor (Daniel and Whitteridge, 1961; Cowey and Rolls, 1974; Van Essen and Gallant, 1994; Dougherty et al., 2003) and measured 11.2 cms in diameter (5.57° of visual angle at the fovea).

#### Data acquisition and analysis

MEG data was acquired at the KIT-Macquarie Brain Research Laboratory, using a 160-channel whole-head KIT system with first-order axial gradiometer sensors (50-mm baseline). Continuous data was acquired at a sampling rate of 1000 Hz and downsampled to 250 Hz prior to further analysis. Fieldtrip (Oostenveld et al., 2011) and SPM 8 (Institute of Cognitive Neurology, London, UK) were used for all analyses. Continuous data were filtered (bandpass 1–45 Hz), epoched around the time of stimulus onset (−400 to 800 ms) and baseline corrected. Artifacts were removed using the Fieldtrip visual artifact rejection method. In this method (as a built-in-SPM function), we can visually observe a summary of all channels and trials. The summary function provides a plot with the variance for each channel and trial. We manually selected the outliers by visually inspecting the data to identify trials and channels affected by eye blinks and movements (~1.9% of trials were removed). Data were co-registered with the individual head shape data and then transformed into a common sensor space (the average sensor space across subjects) using the method described by Knösche (2002) and implemented in Fieldtrip. Average waveforms were then computed for each subject, condition and sensor.

#### Amplitude analysis

The cortical response to visual stimuli shows peak amplitude at about 170 ms (within a time-window of 130–200 ms: Bentin et al., 1996; Rossion and Jacques, 2008; Rossion and Caharel, 2011) post-stimulus onset. In many analyses, the multiple comparisons problem is posed by a large number of sensors in close proximity, leading to a huge number of observations. Restricting the search space prior to inference is one method of circumventing this problem of multiple comparisons but is only valid if an area of interest is defined *a priori*. Such an approach, by excluding observations, necessarily neglects what might well be important data. In our study, we did not specify the space of interest *a priori*. Instead, we used topological inference to search over the entire sensor space for significant responses throughout the time window of −400 to 800 ms. Based on the random field theory (Worsley and Friston, 1995; Worsley, 2003), topological inference for MEG data has been implemented in SPM8 (Kilner and Friston, 2010; Litvak et al., 2011) to correct for multiple statistical comparisons across N-dimensional spaces. Briefly, a 2D topographical representation of the evoked field for each sample of the time dimension across the epoch of interest is created. Here, we created a 64 × 64 pixel image for each of the 300 samples between −400 and 800 ms around the stimulus onset. This allowed us to compare differences in both space and time, while correcting for the family-wise error (FWE) rate across the multiple comparisons. These images were then taken to the second level of the classical SPM analysis and compared using a flexible factorial design with stimulus type (face, place), SF (LSF, HSF), and location (fovea, periphery) as factors. At the second level (group) analysis, we compared the effect of the above factors on the 2D topographies of the event-related fields (ERFs). Significance threshold was set at *p* < 0.05 (FWE-corrected) to determine significant differences between conditions.

#### Latency analysis

We also calculated the M170 latency over occipito-temporal face selective sensors (coinciding with the sensors with maximum amplitude difference for all conditions) for various stimulus conditions. The most face-selective sensors (corresponding to channels 103, 114, 115, 116, 118, 119, 120, 121, 123) were chosen based on the criteria that they had the highest ratio of face to place M170 amplitude in our study (see Figure 4A). These sensors are located over the occipito-temporal region where N170 is shown to be most prominent in a wide variety of studies (Goffaux et al., 2003; Joyce and Rossion, 2005; Halit et al., 2006; Jacques and Rossion, 2009). The selected cluster of sensors coincides with the maximum amplitude difference for other experimental conditions as well. We pooled together both the positive and negative peaks to examine the latency values using a multivariate analysis. The mean of peak latency values from the cluster of selected sensors was then compared using a repeated measures ANOVA for both N1 and P1 (instead of just N1 or P1 latency values), with stimulus type (face, place), SF (LSF, HSF), location (fovea, periphery), and hemifield (left, right) as factors.

### MEG results

**Figure 3:**
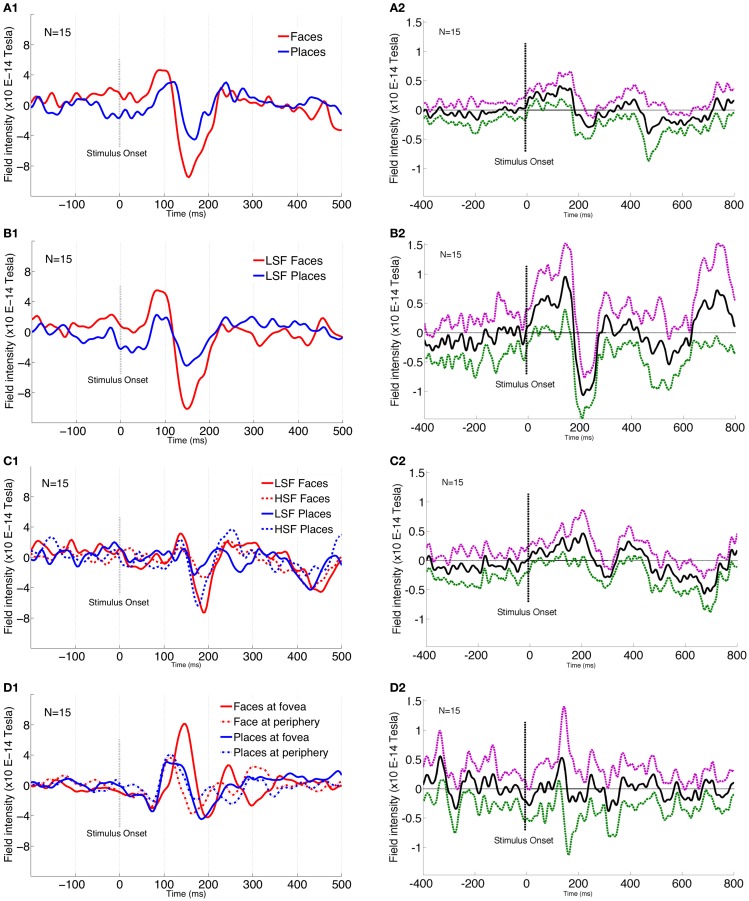
**MEG 170 amplitude plots for various conditions.** 1 plots show the individual conditions where the images are cropped to show between −200 and 500 ms around the stimulus onset. 2 plots show the difference of conditions compared across the entire window of −400 and 800 ms around stimulus onset. In 2 plots, solid black line shows the mean difference while the dotted purple and green lines represent 95% CI in positive and negative directions, respectively. **(A1)** Significant amplitudes for face and place stimuli. Plot is from sensor 118 that corresponds to the highest amplitude difference at 148 ms post-stimulus onset. **(A2)** Plot shows face-place difference across the entire time window (−400 to 800 ms). **(B1)** Significant amplitude difference between LSF faces and LSF Places. Amplitude plot is from sensor 121 that corresponds to the highest amplitude difference at 148 ms post-stimulus onset. **(B2)** Plot shows LSF comparison for face and place conditions across the entire time window (−400 to 800 ms). **(C1)** Significant interaction between spatial frequency and stimulus type. Amplitude plot is from sensor 123 that corresponds to the highest amplitude difference at 160 ms post-stimulus onset. **(C2)** Plot shows HSF comparison conditions for face and place conditions across the entire time window (−400 to 800 ms). **(D1)** Significant interaction between stimulus type and location. Amplitude plot is from sensor 151 that corresponds to the highest amplitude difference at 148 ms post-stimulus onset. **(D2)** Plot shows fovea vs. periphery comparison across the entire time window (−400 to 800 ms).

In agreement with previous research (Liu et al., 2000, 2002; Xu et al., 2005; Harris and Nakayama, 2007), the M170 magnitude for face perception was significantly larger [*F*_(1, 195)_ = 144.89, *p* = 0.001, FWE-corr] than for the perception of places at 148 ms (Figure 3A1). Interestingly, M170 magnitude for LSF faces was significantly larger [*F*_(1, 195)_ = 182.49, *p* < 0.001, FWE-corr] than for LSF places (Figure 3B1). LSF facilitation for faces was stronger than for places, whereas HSF facilitation for places was stronger than for faces, as shown by a significant interaction between SF and stimulus type [*F*_(1, 195)_ = 182.49, *p* < 0.001, FWE-corr] (Figure 3C1) at 160 ms from stimulus onset. *Post-hoc* analysis confirmed that LSF faces elicited significantly larger M170 than HSF faces (*p* = 0.01). Further *post-hoc* paired sample *t*-test results show that HSF places elicited significantly larger M170 than LSF places (*p* = 0.01).

Faces showed a significantly larger M170 when presented at fovea compared to periphery, while this was reversed for places (peripheral places showed a larger M170), as shown by a significant interaction between stimulus type and location [*F*_(1, 195)_ = 172.39, *p* = 0.001, FWE-corr] (Figure 3D1). This was confirmed by a *post-hoc* paired sample *t*-test, showing faces elicited significantly larger M170 for fovea than at periphery (*p* = 0.01), while places elicited significantly larger M170 for periphery rather than at foveal presentation (*p* = 0.01).

To provide a better handle on information content, we examined SF effects across face-place differences for various conditions. Examining the entire time-course, it seems that face-place differences peak around 150 ms (Figure 3A2). The face-place difference for LSF information peaks relatively earlier (Figure 3B2) than that for HSF information (Figure 3C2), suggesting that the critical band for place categorization is likely to be higher than that for face categorization. Figure 3D2 shows the difference contrast for fovea vs. periphery conditions, with the peak around 150 ms.

**Figure 4:**
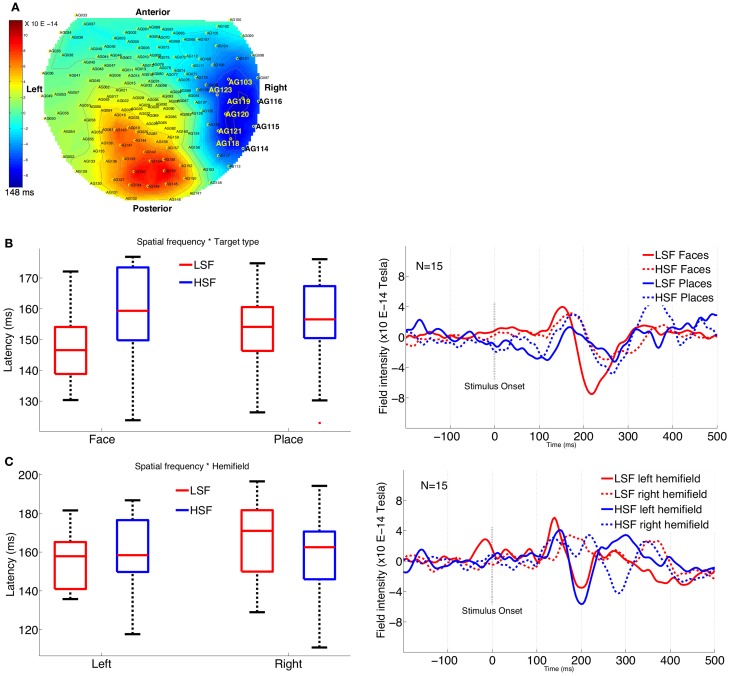
**Plots showing M170 latency across various conditions. (A)** Topographical display of the MEG sensors, shows the cluster of right occipito-temporal sensors selected for latency analysis. **(B)** Interaction graph and plot (from the sensor 121) shows significant latency difference between LSF faces and HSF faces. **(C)** Interaction graph and plot (from the sensor 114) shows significant latency difference for LSF and HSF conditions between left and right hemifield.

SF modulates the latency for M170, with LSF latency significantly earlier than HSF latency, as shown by main effect of SF [*F*_(2, 13)_ = 5.05, *p* = 0.02]. Further, the difference in latency between LSF and HSF faces is significantly greater than the difference between LSF and HSF places as shown by a significant interaction between stimulus type and SF [*F*_(2, 13)_ = 5.57, *p* = 0.01]. *Post-hoc* (Tukey's HSD) analysis confirmed that LSF faces elicited significantly earlier M170 (mean latency: 147 ms) than HSF faces (mean latency: 159 ms) (*p* = 0.004; Figure 4B).

In addition, the latency difference in left vs. right hemifield is significantly greater for LSF conditions than the corresponding differences for HSF conditions, as shown by a significant interaction between SF and hemifield [*F*_(2, 13)_ = 8.67, *p* = 0.004]. *Post-hoc* (Tukey's HSD) analysis confirmed significantly earlier M170 in left hemifield (154 ms) than in the right hemifield (166 ms) for LSF conditions (*p* = 0.01; Figure 4C), while the differences were not significant for HSF conditions (left = 158 ms, right = 156 ms, *p* = 0.76).

## General discussion

In this study, we set out to examine the behavioral and neuromagnetic correlates of face and place categorization in both LSF and HSF conditions at foveal and peripheral locations. In the behavioral study, at both foveal and peripheral locations, subjects were significantly faster in categorizing LSF faces as compared to HSF faces. In contrast, HSF places were faster than LSF places at both locations. This trend was observed across all the three quartiles of the reaction time distribution and is therefore reflective of a complete shift due to SF and stimulus category manipulation. In the MEG experiment, we found a significantly larger M170 for LSF faces compared to LSF places, peaking at a latency of 148 ms. In contrast to the findings by Hsiao et al. (2005), the current MEG results show that LSF faces evoke a significantly larger cortical response than HSF faces. Hsiao et al. used a different range of SF cutoffs (than used here and in other studies) to define their low (<5 cpf) and high (>15 cpf) stimuli.

Previous research has implicated the magnocellular pathway in the rapid detection and categorization of stimuli (Schyns and Oliva, 1994; Nowak and Bullier, 1997; Delorme et al., 1999). Information in LSF faces is carried mainly through the magnocellular channels and may be sufficient to allow accurate detection in the rapid categorization task used in our behavioral study. Significantly faster reaction times for HSF at the fovea than at the periphery are likely due to a higher density of parvocellular channels at the fovea (Lynch et al., 1992). For places, faster reaction times in HSF conditions than LSF conditions indicate the primacy of HSF in place categorization. In a series of fMRI experiments in macaques and humans, Rajimehr et al. (2011) demonstrated preferential activation of the Parahippocampal Place Area (PPA) by HSF information, further delineating the role of HSF in the perception of fine-grained details (edges, borders) in the environment to aid navigation and identification. Recently, Zeidman et al. (2012) also reported significantly greater engagement of the Parahippocampal cortex in space and scene perception with HSF stimuli.

Several EEG studies have reported a larger N170 for face parts compared to configuration (Zion-Golumbic and Bentin, 2007; Daniel and Bentin, 2012) and have further suggested that M170/N170 responds particularly to the eye region (Schyns et al., 2003; Itier et al., 2006). In contrast, as the M170/N170 component is consistently delayed and/or enhanced by face inversion (Bentin et al., 1996; Itier and Taylor, 2004), M170/N170 has been characterized as an index of configural processing (Rossion et al., 2000). Gao et al. (2012) reported that owing to different cortical sources, N170 seems to be more sensitive to individual facial components, whereas the M170 seems more sensitive to face configuration. In our study, we only examined the differences between faces and places due to SF filtering. We did not carry out a whole vs. parts distinction for faces or places here. It is likely that LSF facilitation for configural cues and HSF for featural cues, differ from the holistic information conveyed by the whole vs. parts distinction in these studies.

In this study, we controlled for the energy differences for face and place stimuli. The LSF faces and LSF places were equated for contrast and luminance information (see Figure 1). Similarly, the HSF versions of faces and places had comparable luminance and contrast information (see Figure 1). If the differential responses to LSF and HSF stimuli were due to differential information about the contralateral eye, then there should have been consistent LSF and HSF responses, irrespective of the stimulus category (whether face or place). Instead, we found LSF facilitation for faces and HSF facilitation for place stimuli, i.e., the M170 amplitude was modulated differently by stimulus-type for LSF and HSF information.

Configural information has been manipulated by a variety of means by inversion, scrambling or isolating inner components. Face images filtered to show only the low spatial frequencies convey configural information within the face, while the features of the face are not discernible (Collishaw and Hole, 2000). By contrast, face images filtered to reveal only the high spatial frequencies show both the features and their configuration (Fiorentini et al., 1983). Work by several researchers (Harmon and Julesz, 1973; De Valois and De Valois, 1988; Hughes et al., 1990; Vuilleumier et al., 2003; Goffaux et al., 2005) have shown that LSF information is tuned more toward configural processing while HSF cues facilitate featural processing. More recently, Flevaris et al. (2011) have demonstrated that top-down attentional selection of SF mediates configural and featural processing. In correspondence with the HSF support for part-based information, Gao et al. (2012) also found reduced M170 amplitude for scrambled facial configuration that was insensitive to configural cues.

Further, the cortical response was significantly larger for LSF faces compared to other categories (HSF faces, LSF and HSF places) implying that M170 possibly reflects configural processing. However, we also found significantly larger M170 for HSF places (compared to LSF places). This points to another possibility that M170 might serve as a *diagnostic marker* for various stimuli. As an alternative to the domain specificity vs. domain generality debate surrounding the N/M170 (see Bentin and Carmel, 2002; Carmel and Bentin, 2002; Rossion et al., 2002), it is likely that M170 indexes a category specific response and is subject to modulatory effects of SF information as shown in this study.

In our previous studies, using the visually guided reaching paradigm (Awasthi et al., 2011a), we have shown that while reaching for HSF targets, the early perceptual response is driven by LSF information. In a subsequent study, comparing LSF advantage for faces vis-à-vis places, we reported that LSF information is processed about 95 ms earlier for faces than scenes (Awasthi et al., 2011b). Other researchers have also reported primacy of LSF information in face processing (Goffaux et al., 2003; Goffaux and Rossion, 2006; de Heering et al., 2008). At short latencies, face-selective mechanisms reportedly utilize LSF information to process face information as a whole in just one glance (Richler et al., 2009).

The issue of whether processing metric distances between features also depends on LSF was addressed in a study by Goffaux et al. (2005). In that study, the authors demonstrated that LSF (<8 cpf) facilitated the processing of metric distances (inter-ocular distance and eye height) in a face, while HSF (>32 cpf) facilitated processing of featural information over relational processing. Instead of the first-order vs. second-order configural question, our experiments here aim to examine the categorization of presented stimuli. Moreover, we limited our SF manipulations to specific cut-offs. Both the behavioral and MEG results observed here suggest that LSFs are mostly recruited for processing holistic cues, to a larger extent than for the processing of local metric distances between features, while HSF cues facilitated place categorization. The first-order vs. second-order configural and featural processing is a much larger issue that should be investigated in further studies.

Our finding of foveal preference for faces is consistent with others who argue for eccentricity bias as an organizing principle for the perception of various categories of visual stimuli (Kanwisher, 2001; Levy et al., 2001; Hasson et al., 2002). Recently, Brants et al. (2011) showed that cortical selectivity for *minute distinctions* between visual stimuli is organized at a finer scale while coarse scale selectivity is used for *categorical differentiation*. This further supports the notion that early face processes like face detection and categorization are probably handled by neural mechanisms tuned for rapid processing of coarse information.

Our results concur with previous studies that demonstrate prioritized processing of faces (Farah et al., 1998; Kanwisher and Yovel, 2006). LSF visual information carried through the magnocellular pathway is reported to modulate cortical visual processing in a top down manner (Bar, 2003). This pathway plays a critical role in detecting and directing attention to emotionally salient stimuli in the environment, facilitating communication with conspecifics in the social and survival context. Rapid detection of and orientation toward faces is important during developmental stages, particularly when other specialized cortical regions like FFA and OFC are still maturing.

As we combined a measure of behavioral as well as neural activity, the results obtained from this study are likely to be compelling. However, some potential limitations should be considered before integrating the results of the reaction time and MEG experiments. Due to limitations of screen size in the MEG lab, peripheral locations of stimuli differed (in visual angles) for the behavioral and MEG experiments. As we were interested in measuring the cortical response, peripheral images were enlarged (in accordance with the cortical magnification factor) for the MEG experiment only. Although it is not unusual to concatenate sensors/electrodes within a (usually arbitrarily defined) spatial zone, it should be noted that only selected right hemisphere sensors were included in the latency analysis.

Recently, it has been suggested that high-pass filtering of raw data can distort the resulting waveforms, and induce biases between conditions (Vanrullen, 2011; Acunzo et al., 2012; Rousselet, 2012; Widmann and Schroger, 2012). Although the issue does not affect the results here, we concur with the lower cutoff recommendation by the authors. We used a two-pass or “acausal” filter that has little effect on latencies, especially those after 100 ms (Rousselet, 2012). High pass filtering affects ERP/ERF *onset* latencies wherein, applying an aggressive high pass filter to a step-function can introduce side-lobes that smear the onset in time. This is also not an issue here as we use zero-phase filtering and are examining the latency of the *peak* and not the *onset*.

It is also important to acknowledge a few potential caveats. As reaction time distributions are skewed, the mean is considered a poor estimator of central tendency. In the MEG experiment, some might argue that due to the long stimulus duration, subjects could make saccades, particularly toward peripheral stimuli. We did not use an eye tracker or record EOG which are potential limitations for the interpretation of foveal vs. peripheral effects. As an essential task instruction, subjects were required to maintain fixation at the cross throughout the experimental block. In a recent eye-movement study, Lemieux et al. (2012) showed fixation patterns suggestive of holistic processing at low SFs and featural processing at high SFs, at both encoding and retrieval stages. Further, one might expect that the early differences between conditions show peaks at around 100 ms post-stimulus onset. In the current study however, we did not specify any *a priori* space of interest and used topological inference to search over the entire sensor space for significant responses (Kilner and Friston, 2010; Litvak et al., 2011) across −400 to 800 ms of the time window. After appropriate correction for baseline activity, the first significant difference between conditions only emerged at 148 ms.

In an ERP study, Flevaris et al. (2008) reported that the distinction between faces and cars can be made efficiently using both LSF and HSF information, and argued for relatively automatic access of LSF and HSF during early face categorization. In contrast, Goffaux et al. (2003) compared N170 for spatially filtered images of faces and cars and reported findings similar to our MEG results. However, we used a different set of stimuli (faces and places) in our experiments. The use of only two categories of stimuli is a potential limitation for the conclusions drawn here regarding the differential role of low and high frequencies in face vs. place categorization. It could be the case that the same low frequency band used here for face categorization may be useful for place categorization in a task requiring discrimination between places and another category, say, man-made tools.

The findings presented here have several implications for understanding broader issues in the development of recognition and learning processes. LSF primacy may stem from a variety of reasons (see Hughes et al., 1990). LSF is less prone to image degradation from poor or dim lighting at dawn and dusk or in fog. It also provides better adaptation for visual degenerative conditions like scotoma. The ability to rapidly detect a threat at the periphery, detection of fast moving objects and use of larger receptive fields and thus less neural resources, are all advantages of LSF processing. A magnocellular advantage is essential for the initiation of attention mechanisms in the parietal cortex, facilitating rapid and automatic initial global analysis of the stimulus (Schroeder, 1995; Vidyasagar, 1999, 2004, 2005; Laycock et al., 2007). Evidence presented here suggests that early processing of specific SF information facilitates rapid detection and may encode global stimulus categorization.

### Conflict of interest statement

The authors declare that the research was conducted in the absence of any commercial or financial relationships that could be construed as a potential conflict of interest.
